# Effects from the induction of heat shock proteins in a murine model due to progression of aortic atherosclerosis

**DOI:** 10.1038/s41598-021-86601-8

**Published:** 2021-03-29

**Authors:** Naoya Hashikawa, Masanobu Ido, Yuna Morita, Narumi Hashikawa-Hobara

**Affiliations:** Department of Life Science, Okayama University of Science, 1-1 Ridai-cho, Kita-ku, Okayama, 700-0005 Japan

**Keywords:** Cardiology, Cardiovascular biology, Cardiovascular diseases, Vascular diseases, Aortic diseases

## Abstract

Heat shock proteins (HSPs) are molecular chaperones that repair denatured proteins. The relationship between HSPs and various diseases has been extensively studied. However, the relationship between HSPs and atherosclerosis remains unclear. In this study, we induced the expression of HSPs and analyzed the effects on the development/progression of atherosclerosis in vivo. Remarkably, when HSPs were induced in apolipoprotein E deficient (ApoE^−/−^) mice prior to the formation of atheromas, the progression of atherosclerosis was inhibited; the short-term induction of HSPs significantly decreased the mRNA expression of intercellular adhesion molecule 1 (*ICAM-1*) and vascular cell adhesion molecule 1 (*VCAM-1*) in the aorta. In contrast, the induction of HSPs after the formation of atheromas promoted the progression of atherosclerosis. In fact, the short-term induction of HSPs, after the formation of atheromas, significantly increased the mRNA expression of tumor necrosis factor-alpha, and interleukin 6 in the aorta. Of note, the induction of HSPs also promoted the formation of macrophage-derived foam cells. Overall, these results indicate that HSPs exerts different effects in the context of aortic atherosclerosis, depending on its degree of progression. Therefore, the induction and inhibition of HSPs should be considered for the prevention and treatment of atherosclerosis, respectively.

## Introduction

Atherosclerosis is caused by atheromas, mainly composed of lipid components lining blood vessel walls. This induces an inflammatory reaction, and consequently, blood vessel wall fibrosis and luminal stenosis occur. Therefore, atherosclerosis can develop into ischemic disease, one of the world's leading causes of death^1^. The atherosclerotic lesions are mainly found at the branches and curvatures of large blood vessels. The blood flow in branches and curvatures is unevenly disturbed by the primary cilia of the endothelium^2^. This disturbed flow promotes the phosphorylation of occludin and weakens the tight junctions-mediated barrier function in endothelial cells^3^. Therefore the endothelial cells become permeable to lipoproteins leading to the accumulation of low-density lipoproteins (LDLs) in the intima^4^. These lipoproteins are then modified via different mechanisms, including oxidation by reactive oxygen species (ROS)^5^. Monocyte recruitment from the bloodstream is probably the initial stage in the process of atherosclerotic plaque formation, activated by a regulated multistep process and mediated by chemoattractants and cell adhesion molecules such as vascular cell adhesion molecule (VCAM), and intercellular adhesion molecule (ICAM)^6^. In the intima, monocytes express scavenger receptors, phagocytose modified lipoproteins, and acquire the characteristics of pro-inflammatory macrophages, secreting various inflammatory cytokines [e.g., tumor necrosis factor-alpha (TNF-α), interleukin-1 (IL-1), and IL-6], which, in turn, recruit T cells, and B cells^7^. Altogether, the intimal infiltration and modification of lipoproteins and their uptake mainly by macrophages, and consequent formation of lipid-filled foam cells, initiate atherosclerotic lesion formation^8^.

Apolipoprotein E (ApoE), encoded by a gene with the same name (*APOE*) located on chromosome 19 in humans, is involved in lipid transport and metabolism. *APOE* gene deficiency disrupts lipid transport into the liver, resulting in the development of hyperlipidemia and the progression of aortic atherosclerosis. In fact, ApoE-deficient (ApoE^−/−^) mice are commonly used as a model of aortic atherosclerosis, sharing a greater degree of similarity to the development of atherosclerosis in humans compared to other models^9^. There is a dramatic shift in the profile of lipoproteins in the plasma, with the pro-atherogenic VLDL as the most abundant circulating lipoprotein, similar to that observed in type II hyperlipidemia in humans. Importantly, the spontaneous development of atherosclerotic lesions in several vascular beds, predominantly in the aortic root, aortic arch, and different branch points along the aorta, are observed. When ApoE^−/−^ mice are fed a chow diet, monocyte/macrophage attachment and the subsequent appearance of subintimal lipid-laden macrophages (foam cells) occurs between 6–8 and 8–10 weeks of age, respectively. Moreover, between 15 and 25 weeks of age, macrophage necrosis, and fibrous cap formation occur^10^. Of note, feeding with high-fat and western-type diets accelerates the development of atherosclerosis in ApoE^−/−^ mice^11^.

Molecular chaperones assist other proteins to fold and function correctly. A group of molecular chaperones called heat shock proteins (HSPs) are expressed in response to various stresses, such as heat and ultraviolet light^12,13^. Interestingly, geranylgeranylacetone (GGA), a drug used to treat gastritis and gastric ulcers, induces HSP expression, especially in gastric mucosal cells^14^. Furthermore, various organs, such as the heart, liver, brain, small intestine, and eyes, were also demonstrated to express HSPs in response to different stresses^15–19^. In addition, hyperthermia is another method used to induce HSP expression; particularly, in Japan, hot springs are known for their health maintenance effects. HSPs are involved in the formation of three-dimensional protein structures for the repair of denatured proteins and are grouped into various families depending on their molecular weight^20^. For example; HSP27, an analog of mouse HSP25, is involved in the suppression of apoptosis and remodeling of the actin cytoskeleton^21,22^. Moreover, HSP60, also known as chaperonin, is abundant in prokaryotes and eukaryotes and required for the folding, translocation, and assembly of native proteins^23^. Additionally, HSP72 is involved in anti-inflammatory functions and the refolding of damaged proteins^24^; and HSP90 activates factors related to intracellular signaling^25^. In recent years, it has become clear that HSPs are associated with various diseases, such as neurological disorders and cancer; in fact, the role of HSPs in the context of several diseases has been widely investigated^26–28^. However, the relationship between the progression of atherosclerosis and the expression of HSPs is unclear. Of note, HSPs can be easily induced. Thus the investigation of the effect of HSPs on atherosclerosis may reveal new targets for the prevention/treatment of atherosclerosis.

In this study, we analyzed the preventive or therapeutic effects of the expression of HSPs on the development or progression of atherosclerosis. In addition, we focused on adhesion molecules and inflammatory cytokines in order to elucidate the mechanisms underlying the potential effects of HSPs.

## Results

### The expression of HSPs increases with the formation of atheromas in the aorta in mice

**Figure 1:**
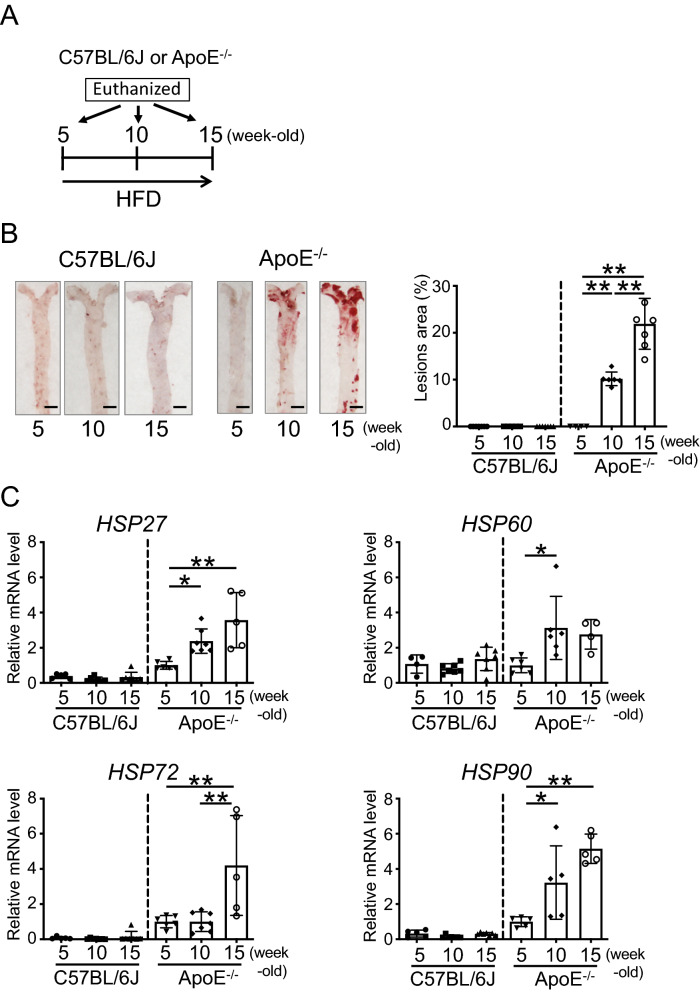
Progression of atherosclerosis and expression of heat shock proteins (HSPs). (**A**) Representation of the experimental timeline. (**B**) Oil Red O staining of the aorta of C57BL/6 J and ApoE^−/−^ mice fed a high-fat diet (HFD) for 0, 5, or 10 weeks (5, 10, or 15 weeks old, respectively) (left). Bar graph showing the quantification of atherosclerotic areas (right). (**C**) *HSP27*, *60*, *72*, and *90* mRNA expression levels in the aorta of C57BL/6 J and ApoE^−/−^ mice fed an HFD for 0, 5, or 10 weeks (5, 10, or 15 weeks old, respectively). Each bar represents the mean ± standard deviation (SD). Significant differences are marked on the chart (*p < 0.05; **p < 0.01). n = 5–7 mice per group. Scale bars = 2 mm.

First, we analyzed atheroma formation, using Oil Red O staining, in C57BL/6 J and ApoE^−/−^ mice fed a high-fat diet (HFD) for 0, 5, or 10 weeks (5, 10, or 15-week-old, respectively). The timeline is shown in Fig. 1A. Atheromas did not form in the aortas of 5, 10, and 15-week-old C57BL/6 J mice as well as in 5-week-old ApoE^−/−^ mice; on the other hand, atheromas formed in the aortas of 10-week-old ApoE^−/−^ mice (approximately 10%), and in 15-week-old ApoE^−/−^ mice, the atherogenic region was widely distributed (approximately 22%) (Fig. 1B).

Next, the mRNA expression of *HSPs* in the aorta was analyzed in 5, 10, and 15-week-old C57BL/6 J and ApoE^−/−^ mice using real-time PCR. The mRNA expression of *HSP27*, *60*, *7*2, and *90* was unchanged in the aortas of 5, 10, and 15-week-old C57BL/6 J mice. Interestingly, in the aortas of 10-week-old ApoE^−/−^ mice fed HFD for 5 weeks, the expression of *HSP27*, *60*, and *HSP90* (P = 0.0450, P = 0.0243, and P = 0.0483, respectively) was significantly increased compared to that in 5-week-old ApoE^−/−^ mice (Fig. 1C; one-way ANOVA). Additionally, in the aortas of 15-week-old ApoE^−/−^ mice fed HFD for 10 weeks, the expression of *HSP27, HSP72*, and *HSP90* (P = 0.0010, P = 0.0090, and P = 0.0008, respectively) was also significantly increased compared to that in 5-week-old ApoE^−/−^ mice (Fig. 1C; one-way ANOVA).

### Induction of the expression of HSPs in the aorta in mice

**Figure 2:**
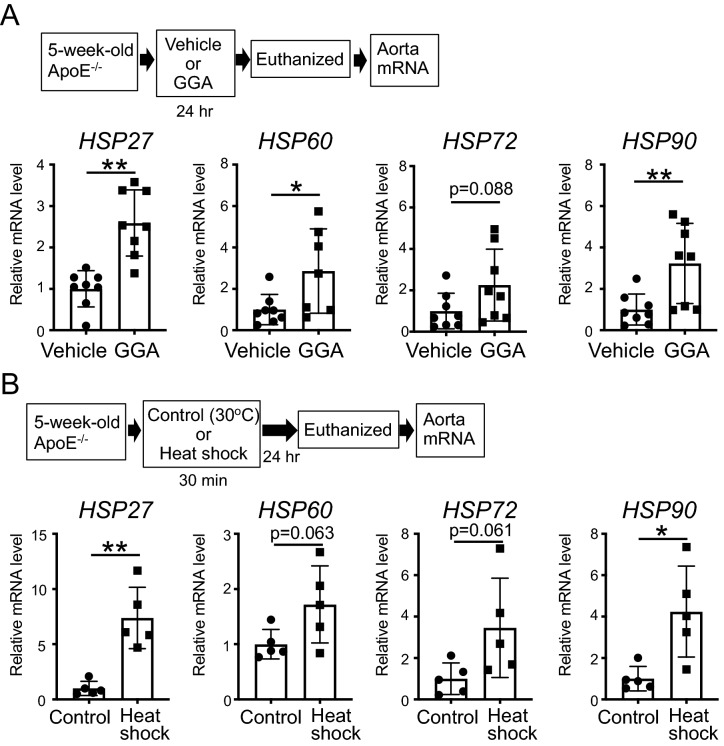
Effect of the induction of heat shock proteins (HSPs) in mice treated with geranylgeranylacetone (GGA) or subjected to heat shock. (**A**) *HSP27*, *60*, *72*, and *90* mRNA expression levels in the aorta after GGA administration. 5-week-old ApoE^−/−^ mice were administered vehicle or GGA (500 mg/kg/day) for 24 h, and total RNA was subsequently extracted from the aorta. (**B**) *HSP27*, *60*, *72*, and *90* mRNA expression levels in the aorta after heat shock treatment. 5-week-old ApoE^−/−^ mice were exposed to a 30 °C (control) or 41 °C (heat shock) bath for 30 min. After 24 h, total RNA was extracted from the aorta. Each bar represents the mean ± standard deviation (SD). Significant differences are marked on the chart (*p < 0.05; **p < 0.01). n = 5–8 mice per group.

As it was suggested that HSPs may be involved in atherosclerosis, we first explored different methods to induce the expression of HSPs in the aorta in vivo*.* Remarkably, ApoE^−/−^ mice orally administered GGA (500 mg/kg/day) for 24 h, demonstrated a significant increase in the mRNA expression of *HSP27*, *60*, and *90* (P = 0.0002, P = 0.0310, P = 0.0089, respectively), and a tendentiously increased *HSP72* (P = 0.0887) in the aorta compared to vehicle-administered mice (Fig. 2A; t-test). In addition, ApoE^−/−^ mice subjected to heat shock using a warm bath at 41 °C for 30 min, also showed a significant increase in the mRNA expression of *HSP27* (P = 0.0011) and *90* (P = 0.0129), and a tendentiously increased *HSP60* (P = 0.0637) and *72* (P = 0.0610) in the aorta after 24 h compared to those exposed to the 30 °C (control group; Fig. 2B; t-test). Therefore, we confirmed that both GGA and heat shock treatments lead to the up-regulation of HSP expression in a similar fashion.

### The induction of the expression of HSPs in ApoE^−/−^ mice before the formation of atheromas prevents atherosclerosis via the reduction of the expression of adhesion molecules

**Figure 3:**
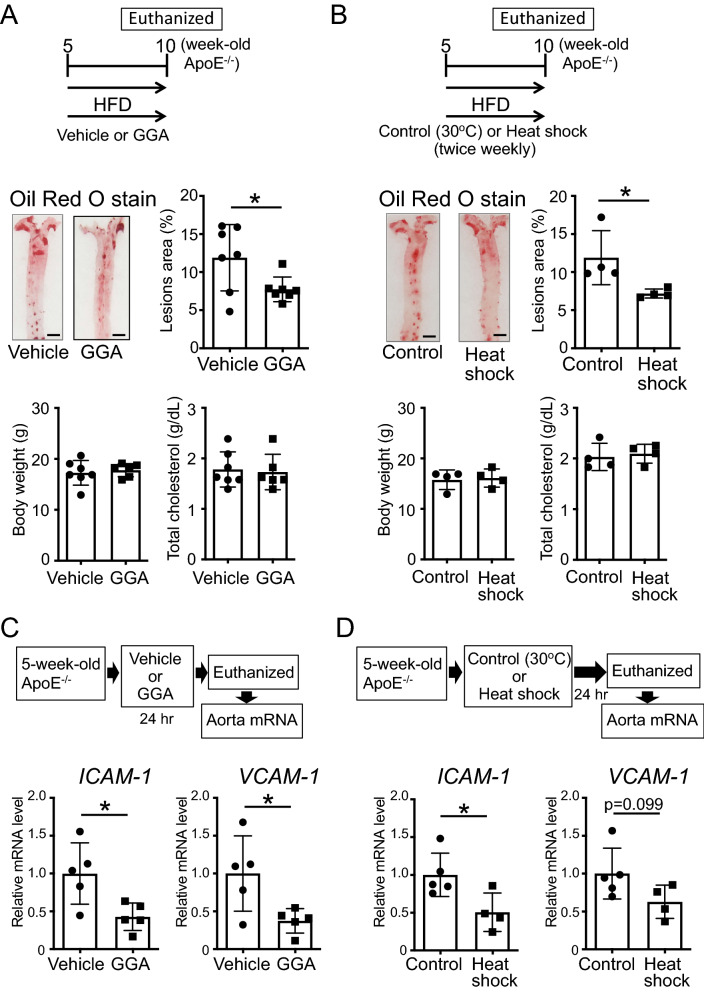
Effect of the induction of heat shock proteins (HSPs) on the prevention of atherosclerosis. (**A**) 5-week-old ApoE^−/−^ mice, with no signs of the formation of atheromas, were administered geranylgeranylacetone (GGA; 500 mg/kg/day) and fed a high-fat diet (HFD) for 5 weeks. Images of the Oil Red O staining of the aorta of ApoE^−/−^ mice are shown. Bar graphs quantifying the atherosclerotic areas, body weight, and total cholesterol levels are also shown. (**B**) 5-week-old ApoE^−/−^ mice, before the formation of atheromas, were treated in a 30 °C (control) or 41 °C (heat shock) warm bath for 30 min, twice weekly, for 5 weeks. At the same time, the mice were fed an HFD for 5 weeks. Images of the Oil Red O staining of the aorta of ApoE^−/−^ mice are shown. Bar graphs quantifying the atherosclerotic areas, body weight, and total cholesterol levels are also shown. (**C**) intercellular adhesion molecule 1 (*ICAM-1*) and vascular cell adhesion molecule 1 (*VCAM-1*) mRNA expression levels in the aorta after GGA administration. 5-week-old ApoE^−/−^ mice, before the formation of atheromas, were administered vehicle or GGA (500 mg/kg/day) for 24 h; total RNA was then extracted from the aortas. (D) *ICAM-1* and *VCAM-1* mRNA expression levels in the aorta after heat shock treatment. 5-week-old ApoE^−/−^ mice, showing no signs of the formation of atheromas, were exposed to a 30 °C (control) or 41 °C (heat shock) warm bath for 30 min. After 24 h, total RNA was extracted from the aortas. Each bar represents the mean ± standard deviation (SD). Significant differences are marked on the chart (*p < 0.05; **p < 0.01). n = 4–7 mice per group. Scale bars = 2 mm.

To clarify whether HSPs have preventive effects on aortic atherogenesis, we induced the expression of HSPs in ApoE^−/−^ mice prior to the formation of atheromas. Importantly, ApoE^−/−^ mice (5-week-old), without atheromas, orally administered GGA (500 mg/kg/day) and fed an HFD for 5 weeks, evidenced a significant reduction in atheroma formation compared to that in vehicle-administered mice (Fig. 3A; t-test, P = 0.0356). Of note, the body weight, and serum total cholesterol levels in GGA-administered mice were similar to those in vehicle-administered mice (Fig. 3A). Similarly, 5-week-old ApoE^−/−^ mice, exposed to 41 °C heat shock treatment for 30 min twice weekly for 5 weeks, showed similar body weight and serum total cholesterol levels but significantly reduced atheroma formation compared those in control animals (Fig. 3B; t-test, P = 0.0401).

Next, to clarify the mechanism behind the HSPs-mediated suppression of atheroma formation, we analyzed the mRNA expression of adhesion molecules, specifically of *ICAM-1* and *VCAM-1*. A significant decrease in the expression of *ICAM-1* (P = 0.0208) and *VCAM-1* (P = 0.0281) was observed in the aorta of 5-week-old ApoE^−/−^ mice, without atheromas, following the oral administration of GGA (500 mg/kg/day) for 24 h (Fig. 3C; t-test). Likewise, 30 min of 41 °C heat shock significantly suppressed the expression of *ICAM-1* (P = 0.0314), and a tendentiously suppressed *VCAM-1* (P = 0.0990) in the aorta of 5-week-old ApoE^−/−^ mice, without atheromas (Fig. 3D; t-test).

### The induction of the expression of HSPs in ApoE^−/−^ mice, after the formation of atheromas, promotes the expression of adhesion molecules and the progression of atherosclerosis

**Figure 4:**
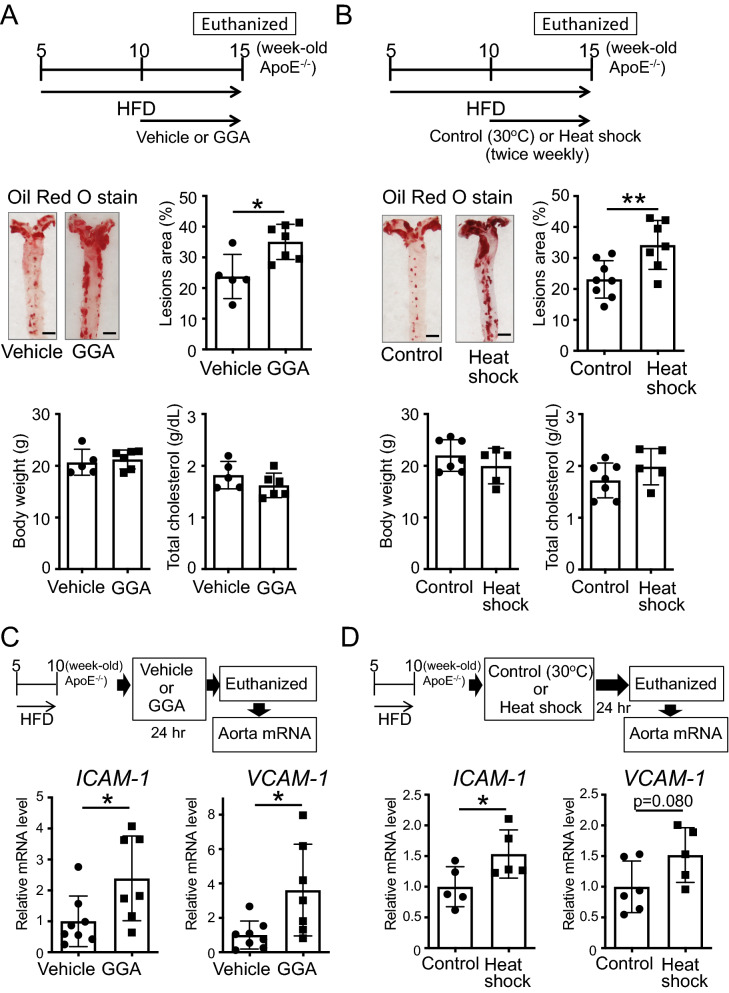
Effect of the induction of heat shock proteins (HSPs) on the progression of atherosclerosis. (**A**) 10-week-old ApoE^−/−^ mice, evidencing the formation of atheromas, were administered geranylgeranylacetone (GGA; 500 mg/kg/day) for 5 weeks. Images of the Oil Red O staining of the aorta of ApoE^−/−^ mice are shown. Bar graphs quantifying the atherosclerotic areas, body weight, and total cholesterol levels are also shown. (**B**) 10-week-old ApoE^−/−^ mice, after the formation of atheromas, were treated in a 30 °C (control) or 41 °C (heat shock) warm bath for 30 min, twice weekly, for 5 weeks. Images of the Oil Red O staining of the aorta of ApoE^−/−^ mice are shown. Bar graphs quantifying the atherosclerotic areas, body weight, and total cholesterol levels are also shown. (**C**) Intercellular adhesion molecule 1 (*ICAM-1*) and vascular cell adhesion molecule 1 (*VCAM-1*) mRNA expression levels in the aorta after GGA administration. 10-week-old ApoE^−/−^ mice, after the formation of atheromas, were administered vehicle or GGA (500 mg/kg/day) for 24 h. Then, total RNA was extracted from the aorta. (**D**) *ICAM-1* and *VCAM-1* mRNA expression levels in the aorta after heat shock treatment. 10-week-old ApoE^−/−^ mice, showing signs of the formation of atheromas, were treated in a 30 °C (control) or 41 °C (heat shock) warm bath for 30 min. After 24 h, total RNA was extracted from the aorta. Each bar represents the mean ± standard deviation (SD). Significant differences are marked on the chart (*p < 0.05; **p < 0.01). n = 5–8 mice per group. Scale bar = 2 mm.

To determine whether the expression of HSPs also ameliorates aortic atherosclerosis after the formation of atheromas, we induced the expression of HSPs in ApoE^−/−^ mice after with formed atheromas. Unexpectedly, ApoE^−/−^ mice, fed an HFD since 5 weeks of age, and orally administered GGA (500 mg/kg/day) from 10 to 15 weeks of age demonstrated a significant increase in aortic atheroma formation (Fig. 4A; t-test, P = 0.0124). As above, their body weight and serum total cholesterol levels were similar to those in vehicle-administered mice (Fig. 4A). Similarly, 10-week-old ApoE^−/−^ mice, exposed to 41 °C heat shock treatment for 30 min twice weekly for 5 weeks, showed similar body weight and serum total cholesterol levels but significantly increased atheroma formation compared to control animals (Fig. 4B; t-test, P = 0.0087).

In addition, 10-week-old ApoE^−/−^ mice, fed an HFD for 5 weeks and orally administered GGA (500 mg/kg/day) for 24 h evidenced a significant increase in the expression of *ICAM-1* (P = 0.0309) and *VCAM-1* (P = 0.0201) in the aorta (Fig. 4C; t-test). Meanwhile, 30 min of 41 °C heat shock treatment also induced a significantly increased *ICAM-1* mRNA expression (P = 0.0478), and a tendentiously increased *VCAM-1* mRNA expression (P = 0.0801) in the aorta (Fig. 4D; t-test).

### The induction of the expression of HSPs activates intraperitoneal macrophage functions

It has been reported that the accumulation of macrophages in the blood vessel walls is greatly involved in atherogenesis^29^. Therefore, the induction of the expression of HSPs in mice with atheromas may promote atherosclerosis progression via effects in the function of macrophages. To clarify this hypothesis, we analyzed intraperitoneal macrophage functions and the expression of inflammatory cytokines in mice, after GGA treatment.

**Figure 5:**
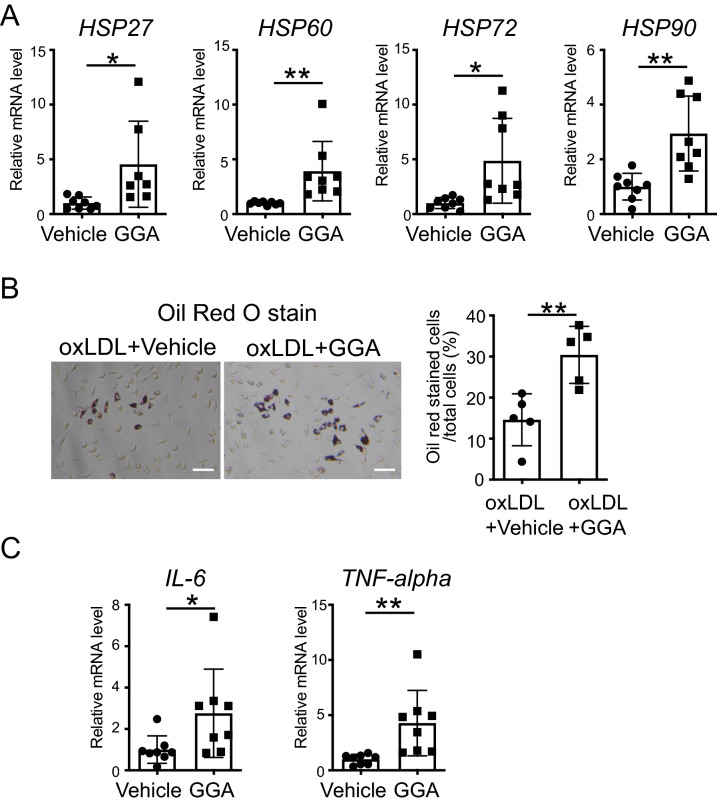
Effect of the induction of heat shock proteins (HSPs) on peritoneal macrophages. (**A**) *HSP27*, *60*, *72*, and *90* mRNA expression levels in ApoE^−/−^ peritoneal macrophages after geranylgeranylacetone (GGA) treatment. Peritoneal macrophages were isolated from 8-week-old ApoE^−/−^ mice. After the cells were treated with vehicle or GGA (10 μM) for 24 h, total RNA was extracted. (**B**) Representative micrographs of Oil Red O staining of peritoneal macrophages (left). Scale bar = 100 µm. Vehicle or GGA (10 μM) was added to the cells, and oxidized low-density lipoproteins (oxLDL) (100 μg/mL) were administered 24 h later. One day later, the cells were fixed and stained. Bar graph quantifying Oil Red O positive cells in peritoneal macrophages (right). (**C**) Interleukin 6 (*IL-6*) and tumor necrosis factor-alpha (*TNF-alpha*) mRNA expression levels in ApoE^−/−^ peritoneal macrophages after GGA treatment. Peritoneal macrophages were isolated from 8-week-old ApoE^−/−^ mice. After the cells were treated with vehicle or GGA (10 μM) for 24 h, total RNA was extracted. Each bar represents the mean ± standard deviation (SD). Significant differences are marked on the chart (*p < 0.05; **p < 0.01). n = 5–8 per group.

First, we observed a significant increase in the expression of *HSP27*, *60*, *72*, and *90* (P = 0.0249, P = 0.0089, P = 0.0142, and P = 0.0020, respectively) in peritoneal macrophages from ApoE^−/−^ mice treated with 10 μM GGA for 24 h (Fig. 5A; t-test).

Additionally, oxLDL was added to peritoneal macrophages 24 h after treatment with vehicle or GGA (10 µM) and their phagocytic activity was examined one day later using Oil Red O staining. Importantly, the percentage of Oil Red O-stained cells and the number of foam cells observed was significantly higher in the GGA-treated versus vehicle-treated groups (Fig. 5B; t-test, P = 0.0055).

It has been reported that inflammatory cytokines are involved in the activation of macrophage function^30^. Therefore, next, we analyzed the effects of the upregulation of HSPs on the expression of inflammatory cytokines in peritoneal macrophages. Interestingly, we found that treatment with GGA (10 µM) significantly increased the mRNA expression levels of inflammatory cytokines, namely *IL-6* (P = 0.0433) and *TNF-alpha* (P = 0.0079), compared to those of the vehicle-treated cells (Fig. 5C; t-test).

### The induction of the expression of HSPs after the formation of atheromas increases the expression of inflammatory cytokines in the aorta in ApoE^−/−^ mice

**Figure 6:**
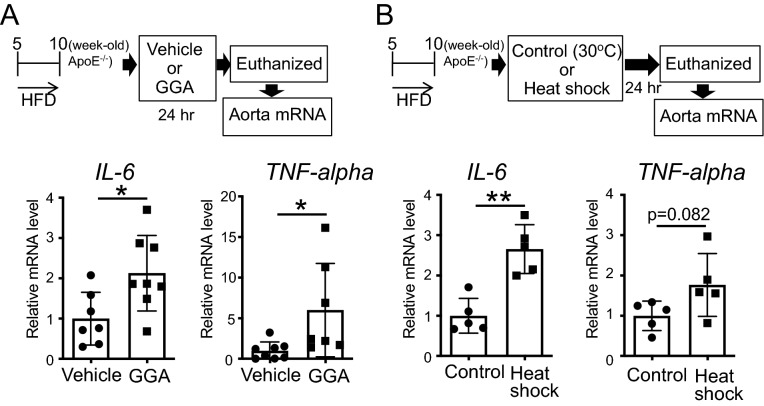
Effect of the induction of heat shock proteins (HSP) on the expression of inflammatory cytokines in the aorta in ApoE^−/−^ mice. (**A**) Interleukin 6 (*IL-6*) and tumor necrosis factor-alpha (*TNF-alpha*) mRNA expression levels in the aorta after geranylgeranylacetone (GGA) administration. 10-week-old ApoE^−/−^ mice, evidencing the formation of atheromas, were administered vehicle or GGA (500 mg/kg/day) for 24 h. Then, total RNA was extracted from the aorta. (**B**) *IL-6* and *TNF-alpha* mRNA expression levels in the aorta after heat shock treatment. 10-week-old ApoE^−/−^ mice, demonstrating the formation of atheromas, were treated in a 30 °C (control) or 41 °C (heat shock) warm bath for 30 min. After 24 h, total RNA was extracted from the aorta. Each bar represents the mean ± standard deviation (SD). Significant differences are marked on the chart (*p < 0.05; **p < 0.01). n = 5–8 mice per group.

To determine the mechanism behind the promotion of atheroma formation due to the induction of HSPs in mice with pre-formed atheromas, we analyzed the expression of inflammatory cytokines in ApoE^−/−^ mice; the aortas of 10-week-old ApoE^−/−^ mice, fed an HFD for 5 weeks, containing atheromas, were isolated following the oral administration of GGA (500 mg/kg/day) for 24 h, and the mRNA expression of *IL-6* and *TNF-alpha* was analyzed. The administration of GGA significantly increased the expression of *IL-6* (P = 0.0195) and *TNF-alpha* (P = 0.0303) in the aorta tissues (Fig. 6A; t-test). Likewise, the exposure for 30 min to 41 °C heat shock significantly increased the expression of *IL-6* (P = 0.0011); additionally, the expression of *TNF-alpha* also tended to increase (P = 0.0823) in the aorta (Fig. 6B; t-test).

## Discussion

In this study, we discovered that when atheromas are scarcely present in the aorta, the induction of HSPs prevents aortic atherosclerosis via the decrease in the expression of adhesion molecules. In contrast, when aortic atheromas are advanced, the induction of HSPs promotes aortic atherosclerosis via the increase in the expression of inflammatory cytokines and the activation of macrophage function. To the best of our knowledge, this is the first report evidencing that the induction of HSPs leads to different effects depending on the degree of the progression of aortic atherosclerosis.

The relationship between several diseases and HSPs has been widely studied^26–28^. In line with this, several reports have evidenced the expression of HSPs during the progression of aortic atherosclerosis. For instance, the increased expression of HSP70 has been observed in atherosclerotic lesions^31^, HSP60 and 70 have been reported to promote lesion progression via the induction of pro-inflammatory and autoimmune responses^32^, and the inhibition of HSP90 appears to prevent disease progression^33^. Conversely, the expression of HSP25 was reported to inhibit aortic atherosclerosis^34^ and increased serum levels of HSP70 were associated with reduced atherosclerotic intimal hyperplasia, and risk of coronary artery disease (CAD)^35^. Therefore, the relationship between HSPs and aortic atherosclerosis remains unknown due to conflicting pieces of evidence. In this study, we induced the expression of HSPs using two different methods, namely GGA administration or heat shock treatment, and analyzed the effects on different contexts of aortic atherosclerosis.

Endothelial cells are greatly involved in arteriosclerosis; aortic atherosclerosis progresses due to the expression of adhesion molecules in endothelial cells^36^. Curiously, here, before the formation of atheromas, the induction of HSPs was found to reduce the expression of adhesion molecules in mice. This is consistent with previous reports showing that HSPs reduce the expression of ICAM-1 in endothelial cells via the inhibition of IκB kinases (IKKs), which activate nuclear factor κB (NF-κB)^37^. In addition, the induction of HSPs before the formation of atheromas, inhibited the progression of atherosclerosis in ApoE^−/−^ mice. These results suggest that HSPs inhibit aortic atheroma progression via the control of endothelial cell functions.

Conversely, and surprisingly, the induction of HSPs after the formation of atheromas promoted the progression of atherosclerosis in ApoE^−/−^ mice. To elucidate the possible reasons behind this, we focused on peritoneal macrophages. It has been reported that the accumulation of macrophages in the wall of blood vessels is greatly involved in atherogenesis^29^. Additionally, the increased phagocytic activity of macrophages was associated with atherogenesis due to the consequent formation of foam cells^38^. Interestingly, here, it was revealed that the induction of HSPs enhanced the phagocytic ability of macrophages in vitro. In addition, we observed that the mRNA expression of the inflammatory cytokines *IL-6* and *TNF-alpha* increased in macrophages. Of note, the induction of the expression of HSPs in vivo also led to the increased expression of inflammatory cytokines (probably macrophage-derived), in the aorta. Moreover, the induction of HSPs, after the formation of atheromas in vivo, also promoted the expression of adhesion molecules in ApoE^−/−^ mice (the complete opposite of that observed before the formation of atheromas). Inflammatory cytokines are known to act on endothelial cells and significantly increase the expression of adhesion molecules^39,40^. These results suggest that the induction of HSPs, following the initiation of atherogenesis, may promote the progression of atherosclerosis via the activation of macrophage function and via the inflammatory cytokines-mediated upregulation of adhesion molecules. However, in the current study, the role of inflammatory cytokines in the HSPs-induced promotion of atherosclerosis remains unclear. Thus, the relationship between HSPs and inflammatory cytokines in atherosclerosis progression needs to be further explored using IL-6 and TNF-alpha knockout mice in the future.

HSPs are generally considered protective since they repair damaged proteins. However, it has been demonstrated that HSPs also protect cancer cells, promote their proliferation, and damage the body. In fact, currently, studies are being conducted on the inhibition of the expression of HSPs for the treatment of cancer^41,42^. Overall, here, we show that the expression of HSPs exerts different effects in the context of atherosclerosis in the aorta, depending on its progression. Prior to the formation of atheromas, the induction of HSPs prevents atherogenesis, however, after the formation of atheromas the upregulation of HSPs leads to the exacerbation of atherosclerosis. Therefore, our findings reveal the differential modulation of the expression of HSPs as a potential approach for the prevention and treatment of atherosclerosis (the induction and inhibition of the expression of HSPs, respectively).

## Methods

### Experimental animals

The animals used in this study were treated in accordance with the Guidelines for Animal Experiments published by the Japanese Association for Laboratory Animal Science, ARRIVE guidelines and the Animal Research Center of the Okayama University of Science, Japan. All experiments were approved by the Animal Care and Use Committee of the Okayama University of Science. Efforts were made to minimize the number of animals used and animal suffering. Mice were housed in a specific pathogen-free room under controlled humidity (50%) and temperature (22 °C) conditions with 12-h light–dark cycles. Male, 4-week-old C57BL/6 J mice were purchased from Shimizu Experimental Animals (Kyoto, Japan) and habituated to a colony for 1 week. ApoE^−/−^ mice (B6.129P2-Apoe tmlUnc, backcrossing at least 10 generations to C57BL/6 J inbred mice; The Jackson Laboratory, Bar Harbor, ME, USA) and C57BL/6 J mice were fed a high-fat diet (HFD; F2HFD1, Oriental Yeast Co., Ltd., Tokyo, Japan) containing 1.25% cholesterol, cocoa butter fat, and 3.0% lard, for either 5 or 10 weeks. In total, 183 mice were used in these experiments (29 C57BL/6 J mice and 154 ApoE^−/−^ mice).

### Induction of the expression of HSPs in mice

The expression of HSPs was induced via the oral administration of GGA or exposure of mice to heat shock. The dosage of GGA was selected based on its effective concentration^26^. GGA (500 mg/kg/day, Tokyo Chemical Industry Co., Ltd.) was emulsified in 5% gum Arabic (FUJIFILM Wako Pure Chemical Corporation) and added to the drinking water; only 5% gum arabic was added to the drinking water in the vehicle group. Additionally, with respect to heat shock induction, mice were immersed in a 41 °C warm bath for 30 min or in a 30 °C lukewarm bath for 30 min (control group). ApoE^−/−^ mice were randomized into the following four groups: vehicle-administered; GGA-administered; 30 °C bath; 41 °C heat shock treatment.

### Analysis of atherosclerosis

The whole length of the isolated aorta was placed in 4% paraformaldehyde and stored at 4 °C. After carefully and completely removing minor branching arteries and fat tissues from its exterior, the aorta was opened longitudinally and stained using Oil Red O (FUJIFILM Wako Pure Chemical Corporation, Osaka, Japan) to identify atheromas. Lesion areas were measured and quantified using the ImageJ software (version 1.52; https://imagej.nih.gov/ij/). All morphological analyses were performed in a blinded manner.

### Measurement of the total cholesterol levels

Blood samples were collected via cardiac puncture after euthanasia, and centrifuged. Sera were stored at − 80 °C until assayed for cholesterol levels. Serum total cholesterol was determined using a cholesterol quantification kit, Cholesterol E-test Wako (FUJIFILM Wako Pure Chemical Corporation) as per the manufacturer’s instructions.

### Cell culture

To isolate peritoneal macrophages, we injected mice with 2 mL of 3% Fluid Thioglycollate Medium (Becton, Dickinson and Company, Franklin Lakes, NJ, USA). Mice were euthanized 2 days later via intraperitoneal injection of sodium pentobarbital (50 mg/kg) and peritoneal macrophages were washed with ice-cold phosphate-buffered saline (PBS) and collected. Cell pellets were suspended in Dulbecco’s modified Eagle medium (DMEM; Thermo Fisher Scientific) containing 10% fetal bovine serum (Thermo Fisher Scientific), 100 U/mL penicillin, and 100 μg/mL streptomycin (Thermo Fisher Scientific). Macrophages were cultured in a humidified incubator containing 5% CO_2_ at 37 °C.

### Macrophage Phagocytosis Assay

GGA was added to the medium at a final concentration of 10 μM one day after plating the peritoneal macrophages (10^5^ cells/well) into 24-well plates. One day later, oxLDL, prepared via incubation of human LDL (Thermo Fisher Scientific) in PBS (containing 20 μM CuSO_4_) at 37 °C for 24 h, and added to macrophages at a final concentration of 100 μg/mL; cells were incubated for 24 h more. Thereafter, cells were washed three times with PBS, fixed with 4% paraformaldehyde for 10 min, stained with Oil Red O, and observed under an optical microscope (Olympus CK40).

### Aorta tissues and macrophages RNA extraction

Mice were euthanized via intraperitoneal injection with sodium pentobarbital (50 mg/kg). To extract RNA from the aorta, the aorta was dissected after euthanasia, and immediately immersed into RNAlater (Thermo Fisher Scientific, Waltham, MA, USA) to inactivate ribonucleases (RNases). The minor branching arteries and fat tissues were subsequently removed. Total RNA was extracted using the RNeasy Fibrous Tissue Mini kit (Qiagen, Hilden, Germany). Additionally, peritoneal macrophages cultured in the presence of 10 μM GGA (Tokyo Chemical Industry Co., Ltd., Tokyo, Japan) for 24 h were aliquoted into RNAlater to enable RNA extraction. Total RNA was extracted using the RNeasy Micro kit (Qiagen, Hilden, Germany).

Following extraction, RNA samples were dissolved into Nuclease-Free Water (Qiagen) and the optical density values of each sample were determined using a NanoDrop Lite Spectrophotometer (Thermo Fisher Scientific). Additionally, we performed reverse transcription according to the manufacturer’s protocol using the Moloney Murine Leukemia Virus (M-MLV) reverse transcriptase (FUJIFILM Wako Pure Chemical Corporation).

### Quantitative analysis using real-time polymerase chain reaction

The reverse transcription reaction mix was used as a template for subsequent real-time polymerase chain reactions (PCR). Real-time PCR was performed using the KAPA SYBR FAST qPCR Kit (NIPPON Genetics Co., Ltd., Tokyo, Japan) and PCR products were analyzed with the Eco Real-Time PCR System (Illumina Inc., San Diego, CA, USA). Primers were designed by the authors (see Table 1 for the detailed sequences) based on the mouse genes coding sequences deposited in the GenBank (NCBI, Bethesda, MD, USA). The data were analyzed using the mean threshold cycle equation. The expression of a gene, along with its relative expression in control samples, was used to calculate the fold change; of note, gene expression was normalized to that of the internal control, glyceraldehyde-3-phosphate dehydrogenase (*GAPDH*). At the end of the real-time PCR run, the Eco Real-Time PCR System generated monophasic melting curves for each PCR product, thereby ensuring amplification specificity.

**Table 1:** Oligonucleotide sequences for real-time PCR amplification.

	Forward (5′–3′)	Reverse (5′–3′)
*HSP27*	AGGAGCTCACAGTGAAGACCAAG	GAGAGATGTAGCCATGTTCGTCC
*HSP72*	TGGTGCTGACGAAGATGAAG	AGGTCGAAGATGAGCACGTT
*HSP90*	AGTCTGGAAGATCCCCAGACCCATG	GGATCATCCTCATCAATACCTAGAC
*ICAM-1*	TAGAGGTGACTGAGGAGTTCGAC	GTAGACTGTTAAGGTCCTCTGCG
*VCAM-1*	GAAGTTCTCCAGCTTCTCTCAGG	CTCCTTCACACACATAGACTCCAG
*IL-6*	CTGGAGTACCATAGCTACCTGGA	GTATCTCTCTGAAGGACTCTGGC
*TNF-alpha*	ACCTTGTCTACTCCCAGGTTCTC	GAGAGGAGGTTGACTTTCTCCTG
*GAPDH*	GCCATTTGCAGTGGCAAAGTGG	GATGGGCTTCCCGTTGATGACA

### Statistical analysis

The GraphPad Prism 7 software was used for statistical analyses. The two-tailed Student’s t-test was used to analyze and compare the difference between two groups. For multiple pairwise comparisons, the one-way analysis of variance (ANOVA) followed by Tukey’s post-hoc analysis was performed. All data are presented as the mean ± standard deviation (SD). Significance is indicated using *p < 0.05 and **p < 0.01.
